# Respiratory Strategies in Relation to Ecology and Behaviour in Three Diurnal Namib Desert Tenebrionid Beetles

**DOI:** 10.3390/insects12111036

**Published:** 2021-11-17

**Authors:** Frances D. Duncan

**Affiliations:** School of Animal, Plant and Environmental Sciences, University of the Witwatersrand, Johannesburg 2050, South Africa; frances.duncan@wits.ac.za

**Keywords:** discontinuous gas exchange, subelytral cavity, metabolic rate, arid habitat, Tenebrionidae, darkling beetles

## Abstract

**Simple Summary:**

The Namib Desert has a large diversity of darkling beetle species. All these beetles are flightless and feed on plant detritus. The aim of this study was to investigate whether the respiration strategies used by these beetles are linked to their ecology and behaviour. Three beetle species, which are all active during the day, in direct sunshine, running across the sand surface, but found in different parts of the desert, were chosen for this study. All three beetle species used intermittent breathing. They held their breath for several minutes and released CO_2_ in pulses. The large beetle species, which runs rapidly on the dune slip face when air temperatures are high, used evaporative cooling to prevent over-heating. The water used in the cooling comes from their respiratory surfaces and is replenished from metabolising food and drinking water droplets left on vegetation after a fog event. The two smaller beetle species, which inhabit the gravel plains, limit the area of the respiratory surface exposed to the atmosphere, which reduces body water loss. These two beetle species are not known to drink water, and thus have a greater need to conserve their body water.

**Abstract:**

The respiratory physiology of three diurnal ultraxerophilous tenebrionid beetles inhabiting either the dune slipface or gravel plain in the Namib Desert was investigated. The role of the mesothoracic spiracles and subelytral cavity in gas exchange was determined by flow-through respirometry. All three species exhibited the discontinuous gas exchange cycles with a distinct convection based flutter period and similar mass specific metabolic rates. There was variation in their respiration mechanics that related to the ecology of the species. The largest beetle species, *Onymacris plana*, living on the dune slipface, has a leaky subelytral cavity and used all its spiracles for gas exchange. Thus, it could use evaporative cooling from its respiratory surface. This species is a fog harvester as well as able to replenish water through metabolising fats while running rapidly. The two smaller species inhabiting the gravel plains, *Metriopus depressus* and *Zophosis amabilis*, used the mesothoracic spiracles almost exclusively for gas exchange as well as increasing the proportional length of the flutter period to reduce respiratory water loss. Neither species have been reported to drink water droplets, and thus conserving respiratory water would allow them to be active longer.

## 1. Introduction

In the Namib Desert, there is a clear link between tenebrionid beetle species and place niche [1,2,3], as well as a lesser link of seasonality and diel activity [3]. The number and diversity of xerophilous tenebrionid species is a unique feature of the Namib Desert [4]. Having xerophilous ancestry has resulted in all the Namib Desert tenebrionid beetles being flightless, and thus possessing a subelytral cavity. This cavity is an airtight space created by the fusion of the elytra [5] and sealing of the fused elytra onto the abdomen via microtrichia fields [6]. The possession of a subelytral cavity and its use in respiration has been important for the success of desert dwelling beetles [7]. The role of the subelytral cavity, into which the elytral spiracles (which consist of the pair of spiracles on the third thoracic segment and abdominal spiracles) open, in respiration has been investigated in both flightless tenebrionid and dung beetles [8,9,10]. Opening the subelytral cavity would release water to the atmosphere and could cause water balance problems for arid dwelling beetles, thus some species only use the mesothoracic spiracle pair, which opens to the atmosphere, for gas exchange. However, the question proposed by Louw [11] as to whether desert animal’s physiology can change so that they are not confined in their distribution in these environments has relevance to the respiratory physiology of tenebrionid beetles.

I have reported on the respiratory strategies of tenebrionid beetles from two other dune field deserts, the Simpson Desert in Australia [10] and the Kalahari Desert in South Africa [8]. These two deserts and the Namib Desert are characterised by dune formations and endemic tenebrionid species [3,12] and have an analogous ecosystem convergence due to the similarities in their climate and geomorphic histories, which shaped the environmental conditions [13]. All the xerophilious tenebrionid beetle tribes in the Namib desert were direct descendants of the fauna from the Kalahari–Karroo–Namaqualand biogeographical system [4] supported from investigations of the phylogeny of psammophilous tribes; Zophosini and Adesmiini [14]. Thus, the tenebrionid beetles in these two deserts are xerophilous by inheritance, whereas the ancestral Simpson Desert tenebrionid beetles probably evolved in mesic forest areas [15] and, due to continental drying producing the current arid dune environment, drove the evolution of xerophilic genera [12]. Both the Simpson Desert and Kalahari Desert tenebrionid beetles mainly use the pair of mesothoracic spiracles as the site of gas exchange, but only the Kalahari tenebrionid beetles use the form of respiration called discontinuous gas exchange cycles [8,10].

Namib Desert tenebrionid beetles have been shown to use discontinuous gas exchange cycles in all the species studied [16], although the role of the mesothoracic spiracles and subelytral cavity in gas exchange is unknown. This form of respiration is characterised by the simultaneous intermittent opening and closing of the spiracles [17,18]. Basically, three periods are involved and referred to as; closed, flutter and burst periods (for a more detailed description of the three periods see [19,20,21]). The closed period being the closure of all the spiracles and no water is lost through the spiracles. During the flutter period, the spiracle valves open and close rapidly to change the size of the spiracle opening, oxygen enters via convection and small amounts of CO_2_ exit. This enables the replenishment of the O_2_ being consumed while reducing the outward movement of CO_2_ and water. In the burst or open period, the spiracles open fully to enable the rapid egress of the accumulated CO_2_ in the insect to the atmosphere and uptake of O_2_. While the spiracles are open, there is some loss of water from the tracheal system as well.

The discontinuous gas exchange cycles contribute to reduce respiratory water loss, specifically if the closed and flutter periods are lengthened, and therefore could be important in reducing overall water loss in a xeric environment [22]. However, not all desert dwelling tenebrionid beetles use this form of gas exchange. Respiratory water loss can also be reduced by using convection to transport of CO_2_ out of the insect [19]. Discontinuous gas exchange cycles may not be the only or main respiratory adaptation to arid environments and, as suggested [23], the mechanisms of gas exchange also need investigation. In previous studies, I have shown that both arid adapted tenebrionid [8,10] and scarab beetles [9] use greater amounts of convective gas transport and the mesothoracic pair of spiracles predominately as the site of the gas transport (see Figure 1 in [24]). Using only one pair of spiracles reduces the respiratory surface area exposed to the environment, thus reducing water loss.

In this study, I looked at the respiratory physiology of Namib Desert tenebrionid beetles to determine whether there is a link between the different gas exchange strategies and the ecology and behaviour of the species. Three beetle species that have high abundance, inhabit extremely arid sub-habitats, viz. sandy dunes and gravel plain [2], and feed mainly on plant detritus [1,25] were chosen. *Onymacris plana* is the dominant beetle species on the dune slipface and is considered a dune specialist [26]. This species can be seen running on the open sand during the day at surface temperatures between 20 and 50 °C, has one of the highest recorded upper lethal temperature of 50–51 °C [27] and the lowest rate of water loss per unit weight measured for any Namib Desert beetle [28]. The other two species inhabit the gravel plain which has the lowest quantity of detritus [2]. *Zophosis* (*Calosis*) *amabilis* is a small oval shaped beetle that forages in the non-vegetative part of the gravel plain [25]. This species is active mainly at midday when surface temperatures range between 45–50 °C [29]. It is considered the most adapted arid beetle as it has exceptional tolerance to heat and low humidity [25] and is unusually drought tolerant, as indicated by being the only species recorded throughout the deepest drought [30]. *Zophosis amabilis* has a relatively impermeable cuticle and was found to have the lowest rate of water loss per unit surface area of the Namib beetles [28]. The other gravel plain species, *Metriopus depressus*, lives in the rocky outcrops [25] and has been observed to retreat under rocks when conditions get too harsh.

Reduction of respiratory water loss would be important to all three species as they are exposed to high temperatures and low humidity. The aim of this study was, therefore, to investigate the gas exchange characteristics, namely flutter and burst periods of the discontinuous gas exchange cycle, and the site of gas exchange in these species. Due to their arid habitat and diurnal activity, it was predicted that the beetles would increase the length of the flutter period, use convection based transport of CO_2_ and maintain a sealed subelytral cavity. Differences in these respiratory characteristics and mechanisms would be linked to the beetle species place niche and behaviour.

## 2. Materials and Methods

### 2.1. Beetles

The beetles were collected while they were actively foraging in the dunes or gravel plain near the Gobabeb Namib Research Institute (23°32′ S, 15°02′ E) in the Namib Desert, Namibia. *Onymacris plana* (Péringuey) (Tribe Adesmiini) was abundant on the dune slipface while *Metriopus depressus* (Haag) (Tribe Adesmiini) and *Zophosis* (*Calosis*) *amabilis* Deyrolle (Tribe Zophosini) were collected from the gravel plain. Detailed descriptions of the Namib Desert habitat and the various sub-habitats are given in [31,32] and details of the habitat in which these species are found in [2]. The beetles were collected and exported with permission from the Namibian Ministry of Environment and Tourism, Directorate of Scientific Serves. Permission was obtained from the South African Department of Agriculture to import these beetles which were housed at the insectary at the University of the Witwatersrand. The beetles were housed in 10-L terraria with a layer of Namib Desert sand and fed dry oats and fresh cabbage leaves. The insectary was kept at 25 °C and on a LD 14:10 h photoperiod. The beetles survived for several months under laboratory conditions.

All three species are flightless with sealed elytra and active during the day. Only *O. plana* has a morphological distinct sexual dimorphism. The males have a flatter wider elytral case than the females, which have the typical dome shape. In this species, the males and females were measured separately to investigate whether the shape of the subelytral cavity had an influence on their gas exchange spiracle use and patterns. Both *M. depressus* and *Z. amabilis* beetles have a dome shaped elytral case, and the sexes could not be separated.

### 2.2. Respirometry

A flow-through respirometry set-up which enables the CO_2_ emission from the mesothoracic spiracles to be measured separately and simultaneously with the CO_2_ emission from the elytral case was used to measure gas exchange in these beetles. The set-up has been previously used for dung beetles and tenebrionid beetles [8,9,10,24]. A single beetle was placed in a respirometry chamber with a latex sheet (dental dam, 0.02 mm thick) separating the chamber into two halves each of approximately 100 mL each in volume. The beetle was pushed through a hole in the latex sheet so that the sheet separated the anterior region of the beetle from the elytral case. The latex sheet made an airtight connection with the body of the beetle at this junction and was kept in place using rubber cement. The CO_2_ emission from the split beetle was measured simultaneously and separately from each chamber. Basically, outside air was drawn through H_2_O and CO_2_ scrubbers (drierite/ascarite) to remove water vapour and CO_2_ from the air at a rate of 50 mL min^−1^ controlled by separate Supelco mass flow controllers. The CO_2_ from the air drawn over the beetle was measured by an individual Licor CO_2_ analyser (either LI-6251 or LI-7000) with a resolution of 0.1 ppm. The pressure in each chamber was monitored continuously using a manometer attached to each chamber to ensure that there were no pressure differences which could alter the results. If any changes in pressure occurred during the measurements, these results were discarded. On placing the beetle in the respirometry chamber and before each measurement, a bolus of O_2_ was injected into the air stream through one half of the respirometry chamber. A Sable System O_2_ analyser (FC-1B) was then used to measure the air from the other half of the respirometry chamber to determine whether there was any leakage between the chambers. To ensure that possible undetected small differences in pressure between the two halves of the chamber were neutralised, towards the end of the measurements the beetle’s position in the chamber was reversed and the resulting CO_2_ traces and CO_2_ emission values compared. Individual beetles were weighed before and after each measurement on a Libror AEG-455M balance (accuracy 0.1 mg).

The beetles were measured during the night for approximately 8 to 10 h. Readings of the volume of CO_2_ emitted were taken every 2 s and recorded using the Expedata (Sable Systems) computerised data acquisition software. Only those CO_2_ traces which showed discontinuous gas exchange cycles, which is an indication that the beetle was not active [21], were used in the analysis. Baseline measurements with the empty chamber separated with a solid piece of latex were taken before and after each beetle measurement. These measurements were used to determine the baseline zero and to correct for analyser drift. CO_2_ output was corrected to standard temperature and pressure (STP) and then converted from part per million to ml h^−1^ or µL h^−1^ ($\dot{V}$_CO2_) using the recommended equations [33,34] to determine metabolic rate.

Various parameters of the discontinuous gas exchange cycle were analysed as these depend on the insect state and environmental influences. The frequency (=burst frequency) of the discontinuous gas exchange cycles is the number of peaks of CO_2_ per hour, and one complete discontinuous gas exchange cycle is the duration of one cycle consisting of the three periods viz. closed, flutter and burst. The mean rate of CO_2_ emission in µL h^−1^ is the mean value of CO_2_ over several discontinuous gas exchange cycles and for a period of at least one hour. For the analysis of the flutter and burst period, which are important to determine differences between species in response to their arid environment, at least 20 to 60 discontinuous gas exchange cycles were measured for each beetle, and the average was taken as a single sample. Integration of the area under the CO_2_ peaks against hours gave the emission volume for the flutter and burst periods, and their duration was measured in seconds. Data are presented as mean ± standard deviation with the sample size (N) representing individual beetles to ensure that all individuals contributed equally to the data set. Unless otherwise noted statistical comparisons were made with one-way analysis of variance. The regression analysis to determine the contribution of the mesothoracic spiracles and elytral case to CO_2_ emission as a function of total CO_2_ emission was calculated using the least squares method. The data was analysed using Python programme, SciPy for statistical analysis and the graphs were drawn using Matplot.

## 3. Results

*Onymacris plana* was significantly larger than the other two species (*F*_2,28_ = 35.28, *p* < 0.0001), but there was no difference in the mass of the males and females (Table 1, *t* = −0.486, *p* = 0.317). A comparison of the mass of the other two species showed a significant difference (*t* = 16.7, *p* = 0.001). The metabolic rate measured as CO_2_ emission is given for the three beetle species in Table 1. The mass-specific metabolic rate of the male and female *O. plana* did not differ significantly (*t* = 0.899, *p* = 0.192), so the results from these beetles were combined (average mass specific metabolic rate = 115.5 ± 42.6 µL h^−1^g^−1^) to compare the three species. Using ANOVA there was no significant differences between the mass specific metabolic rates of the three beetle species (Table 1, *F*_2,28_ = 3.14, *p* = 0.06).

**Figure 1:**
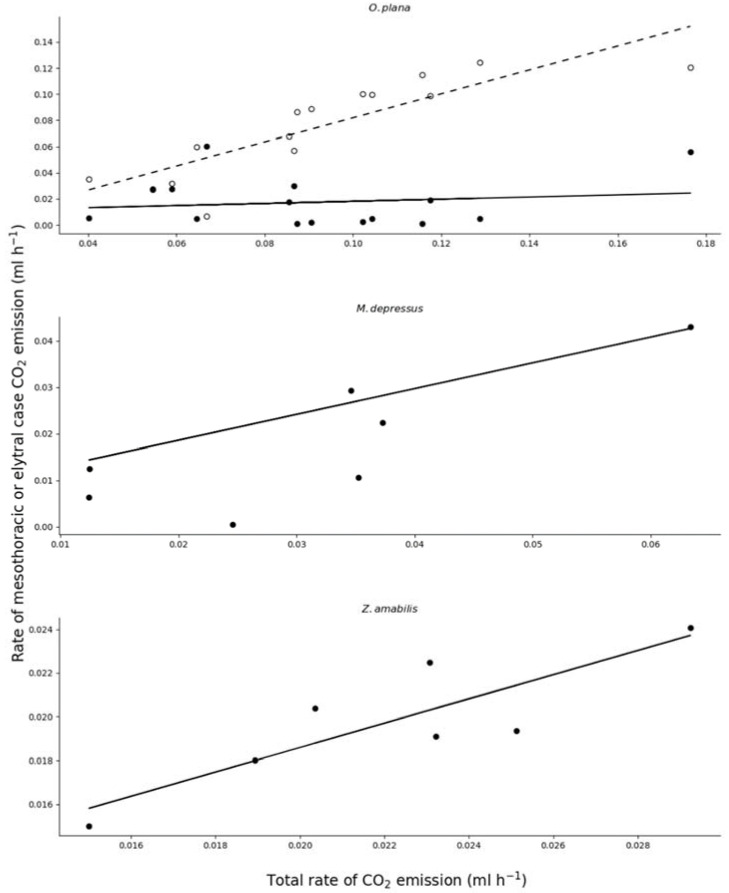
Comparison of the CO_2_ emission rates from the mesothoracic spiracles (●) as a function of total CO_2_ emitted for three Namib Desert tenebrionid beetles. Only *O. plana* has the CO_2_ emission rate from the elytral case (○) as a function of total CO_2_ emitted as the other two species had two or more individuals in which there was no CO_2_ emitted through the elytral case. The regression equation for each species is given where m = mesothoracic spiracle $\dot{V}$_CO2_, *el* = elytral case $\dot{V}$_CO2_, *t* = total $\dot{V}$_CO2_. For *O. plana*: *m* = 0.01 + 0.69*t*, r^2^ = 0.14, *p* = 0.6; *el* = −0.01 + 0.92*t*, r^2^ = 0.85, *p* < 0.0001. For *M. depressus*: *m* = −0.004 + 0.69*t*, r^2^ = 0.83, *p* = 0.022. For *Z. amabilis*: *m* = 0.007 + 0.56*t*, r^2^ = 0.86, *p* = 0.014.

The rate of CO_2_ emission through the mesothoracic spiracles and that measured from outside the elytral case are given for the three species in Table 1. None of the *O. plana* beetles used the mesothoracic spiracles exclusively, but one *M. depressus* beetle and two *Z. amabilis* beetles emitted CO_2_ through the mesothoracic spiracles only. Both *M. depressus* and *Z. amabilis* used the mesothoracic spiracles as the preferred site of CO_2_ emission (over 50%), whereas *O. plana* tended to allow more emission through the elytral case (Table 1). Figure 1 shows the increase in the rate of CO_2_ emissions from the mesothoracic spiracles and elytral case as a function of increasing metabolic rate for the combined male and female *O. plana* data and the increase in the rate of CO_2_ emission from the mesothoracic spiracles in response to increasing metabolic rate for *M. depressus* and *Z. amabilis*. Not only did *O. plana* emit most CO_2_ from the elytral case, but these beetles also increased emission from the elytral case as their metabolic rate increased while the other two species only increased the emission from their mesothoracic spiracles in response to increasing metabolic rate (Figure 1).

**Figure 2:**
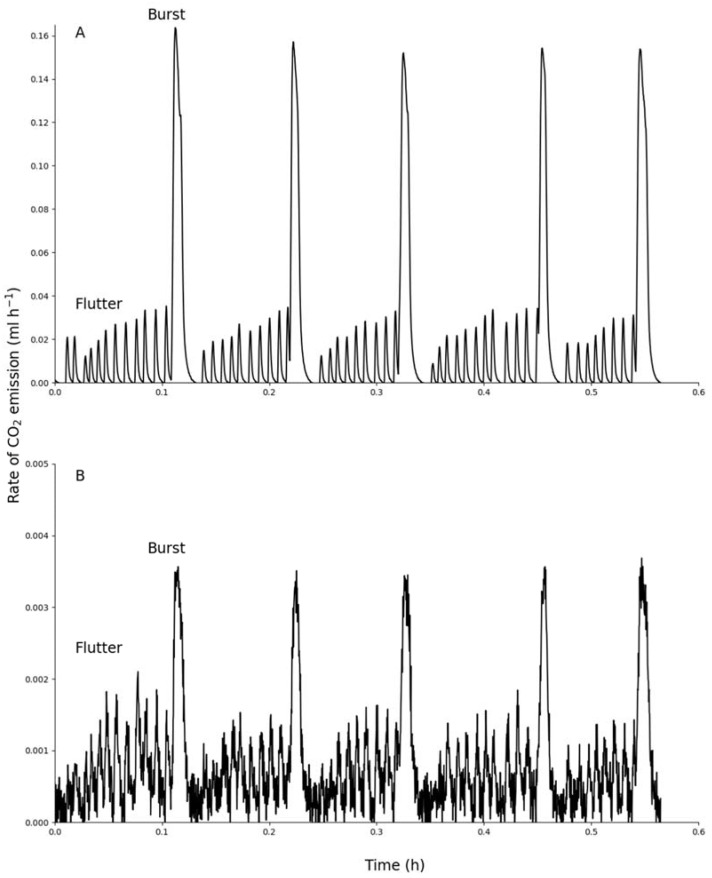
Recording of CO_2_ emission from the mesothoracic spiracles (**A**) and elytral case (**B**) simultaneously in *Z. amabilis* (mass = 0.16 g).

**Figure 3:**
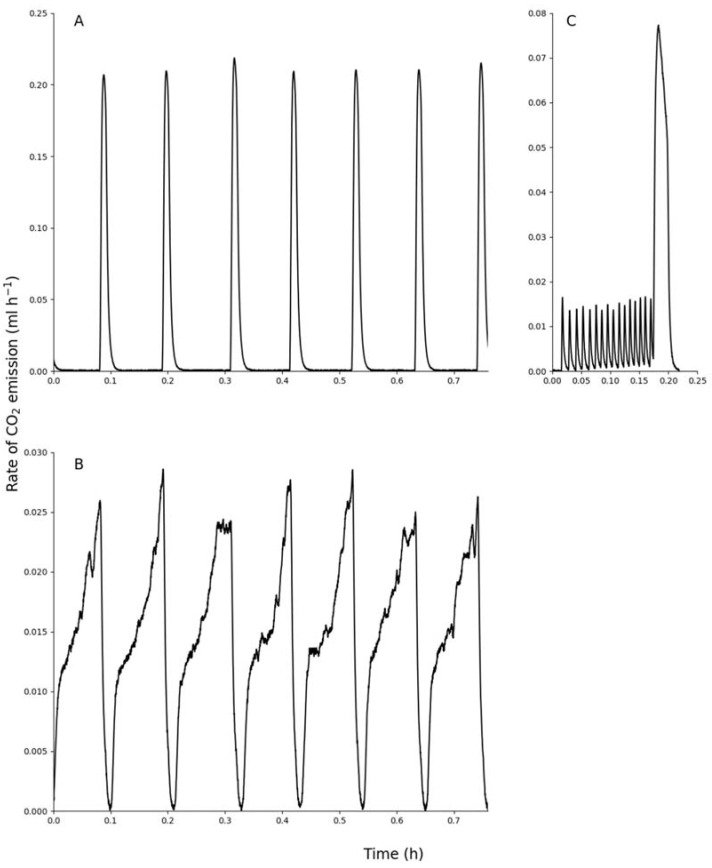
Recording of CO_2_ emission from the mesothoracic spiracles (**A**) and elytral case (**B**) simultaneously in *M*. *depressus* (mass = 0.21 g). For comparison, (**C**) shows one discontinuous gas exchange cycle from the mesothoracic spiracles of a beetle exhibiting the flutter period (mass = 0.18 g).

All three species exhibited discontinuous gas exchange cycles, but the frequency of the cycles differed, with *Z. amabilis* having significantly more cycles per hour than the other two species (Table 1, *F*_2,28_ = 6.09, *p* = 0.006). The frequency of discontinuous gas exchange cycles was not significantly different between the male and female *O. plana* (*t* = −0.178, *p* = 0.43). The discontinuous gas exchange cycles from all three species exhibited intermittent pulsation in the flutter period (Figure 2) as previously described [16]. However, there was a difference in the discontinuous gas exchange cycles in some *M. depressus* beetles. Four beetles from this species did not exhibit the flutter period in all the recordings. These beetles exhibited only a burst period from the mesothoracic spiracles and a combined flutter and burst period from the elytral case (Figure 3). In Table 2, the burst duration given is the length of the mesothoracic burst periods, while the burst volume (µL g^−1^) for the elytral case in these beetles was determined from the extended burst period observed.

The measurements of CO_2_ emission characteristics of the flutter and burst periods are shown in Table 2. These values were obtained by using an average of 44 cycles per beetle for *O. plana*, an average of 22 cycles per beetle for *M. depressus* and an average of 54 cycles per beetle for *Z. amabilis*. During the closed periods there was negligible CO_2_ emission. About 20% of the total CO_2_ emitted per cycle was during the flutter period in *O. plana* and *Z. amabilis*. The flutter period was only observed in three *M. depressus* beetles. In these beetles 43% of the total CO_2_ was emitted through the flutter period. The ratio of emission from the mesothoracic spiracles to that of the elytral case was greatest in *Z. amabilis* in both the flutter and burst periods. In both the male and female *O. plana* the flutter period duration was longer than the burst period duration and a greater percentage of the discontinuous gas exchange cycle duration. The flutter period exhibited by *Z. amabilis* was greater than half of the discontinuous gas exchange cycle duration, while the percentage of the discontinuous gas exchange cycle duration occupied by the burst period was similar to that measured in *O. plana*. In *M. depressus*, the burst period lengths from the mesothoracic spiracles were short and only comprised 19% of the discontinuous gas exchange cycle duration whereas, when including the longer burst periods measured from the elytral case of some of the beetles, this percent duration increased to 30%. In all the CO_2_ emission traces, there was no evidence of ventilation during the burst periods from either the mesothoracic spiracles or elytral case (Figure 2) except in four *M. depressus* beetles (Figure 3) which exhibited limited ventilation in the combined flutter and burst periods.

## 4. Discussion

The three tenebrionid Namib Desert species exhibited flexibility in their gas exchange mechanisms, indicating variation in the respiratory system related to the ecology of the beetle. This implies that the beetle’s respiratory physiology does not limit their distribution and abundance, and thus distribution and abundance of Namib Desert tenebrionid beetles [2,30] are probably due to other ecological factors.

The metabolic rates reported in this study are similar to those reported for other arid adapted tenebrionid beetles [8,10,16,26,35]. Thus, these species have not lowered their metabolic rates compared to other desert species. Interestingly, the temperatures that Namib Desert beetles experience within their habitat range do not have a significant influence on their metabolic rate [26]. Temperature is known to influence metabolic rate with increasing temperature resulting in increasing metabolic rates [23]. However, for Namib Desert tenebrionid beetles, the ecologically relevant temperatures for diurnal beetles (20 to 40 °C) do not significantly change their metabolic rates [26]. This enables these beetles to be active at the high desert temperatures without significantly increasing their metabolic rates and thus requiring increased food resources.

The two beetle species inhabiting the gravel plain, *M. depressus* and *Z. amabilis*, used the mesothoracic spiracles as the main site for gas exchange, but *O. plana* expelled most of the CO_2_ through the elytra. This species, unlike other arid adapted flightless beetles, has a porous subelytral cavity. The microtrichia fields are probably not tightly joined, and thus gas exchange can occur through the subeltyral cavity and at the site of the abdominal spiracles. Approximately 12.5% CO_2_ emission would be expected through the mesothoracic spiracles if all eight pairs of spiracles are being used for gas exchange. As 13.3% of the total CO_2_ emission is via the mesothoracic spiracles in male *O. plana* beetles, it can be assumed that all the spiracles are used for gas exchange as found in a flying dung beetle [9]. In the females, a greater percentage of CO_2_ was emitted through the mesothoracic spiracles (33%), which could be attributed to the difference in shape of their elytra or could be normal biological variation. The male has a flattened elytral cavity which may assist with rapid CO_2_ expulsion. The importance of the abdominal spiracles for O_2_ uptake in *O. plana* has been previously described [36], where it was shown that abdominal pumping corresponded with peaks of O_2_ uptake. These results show that not all the Namib Desert tenebrionid beetles use their abdominal spiracles for gas exchange. In those that predominantly use the mesothoracic spiracles for gas exchange, the abdominal pumping may be creating anteriograde airflow as demonstrated for a flightless dung beetle [24].

*Onymacris plana* is a large beetle and runs at high speeds (90 cm s^−1^) over the desert sand [37], but at the same time retains its body temperature within a narrow range (35.8–38.9 °C for males and 35.0 to 37.9 °C for females [37]). Evaporative cooling by losing water through their spiracles [28] and forced convection [38] have been proposed as the mechanisms used to cool the beetles while running. These beetles rapidly lost water when their spiracles were kept open by placing them in a high CO_2_ environment [28]. Thus, having a porous subelytral cavity will assist in evaporative cooling. While running the beetle also produces metabolic water which is estimated to replace a third or half that lost through transpiration [39]. In this way *O. plana* can extend its activity periods into the potentially lethal ambient temperatures in direct solar radiation when few other animals in the habitat are active. Remaining active rather than having to bury under the sand to reduce body temperature allows the beetles more time to forage and seek for mates.

The two gravel plain dwelling diurnal beetle species exhibited a similar pattern of discontinuous gas exchange cycles as described for the diurnal dune sea and river bed dwelling beetles [16]. Although there is considerable debate as to what the evolutionary drivers for the development of the discontinuous gas exchange cycles are [23], several studies have shown that the use of these cycles does reduce respiratory water loss [18,20] and many arid adapted insects used this form of gas exchange [17]. All three species had the same percentage duration of the burst period of the cycles (30%), which is similar to that found in other flightless beetles [8,32]. A shorter burst or open period will reduce the amount of respiratory water loss. Cockroaches exposed to low humidity reduced the duration of the open period [40] compared to those exposed to high humidities. During the burst period, the spiracles open and CO_2_ exited via diffusion. This period needs to be limited, as convective transport of tracheal gases is required to reduce respiratory water loss [19]. The beetles in this study reduced their respiratory water loss by extending the length of the convection based flutter period within the discontinuous gas exchange cycles [21] and only fully opened the spiracles when necessitated by the increase in internal partial pressure of CO_2_. The flutter period exhibited by the Namib Desert beetles and other desert *Zophosis* species [35] has a greater convection component than the flutter periods observed in other insects using discontinuous gas exchange cycles [16]. By increasing the length of the flutter period as a percentage of the length of a single discontinuous gas exchange cycle, as shown by *M. depressus* and *Z. amabilis*, the beetles can reduce their respiratory water loss. The percent of total CO_2_ emitted during the flutter period was similar to the value of 24% as determined for several other Namib Desert tenebrionid species [16]. The most variable discontinuous gas exchange cycles were shown by *M. depressus*. In some cases the spiracles opening into the subelytral cavity acted differently to the mesothoracic spiracles. The discontinuous gas exchange cycles from the mesothoracic spiracles showed a longer closed period and short burst period only, while those in the subelytral cavity exhibited a short closed period and a combined flutter and burst period with some convective transport of gases. The trigger for these different forms of discontinuous gas exchange cycles is unknown, but these results do show that the discontinuous gas exchange cycles can be changed in this species with the mesothoracic spiracles opening and closing independently of the spiracles opening into the subelytral cavity.

The diversity of tenebrionid beetles found in the Namib Desert could be partially explained by their flexibility in the use of their spiracles. The dune specialist species, *O. plana*, has a competitive advantage by running over the hot sand when temperatures are too high for other species including predators. For these reasons, this species does not show the respiratory water saving strategies as shown for other tenebrionid beetles, which begs the question as to whether this species is water stressed. *Onymacris plana* is known to be an active fog-harvester [40] and drinks the droplets of water left on vegetation after a fog event, enabling the beetle to rehydrate. Fog-harvesting is limited to only a very few of the Namib Desert tenebrionid species [41]. In addition, this beetle species is also able to produce a waxy bloom to prevent water loss [42], which is also limited to a few species, and is efficient in metabolising fat to maintain its body water during desiccation [43]. Running rapidly increases gain of metabolic water [39], along with increasing encounters with food to replenish metabolic lipids. The lowest rate of water loss per unit weight has been measured for *O. plana* [28] with respiratory water loss contributing approximately 70% of the total evaporative water loss [44] with a greater percentage of total water loss from the subeltyral cavity compared to the arid adapted tenebrionid beetle, *Phrynocolus petrosus* [45]. Having access to free water, albeit intermittently [40], metabolising lipids for metabolic water, and several physiological mechanisms to cope with desiccation, the reduction of respiratory water loss from the subelytral cavity is probably not essential for *O. plana*.

In contrast, reducing respiratory water loss might be important to the success of *Z. amabilis* in the gravel plain. This species also has remarkable tolerance to heat and low humidity [27]. This is a small beetle and thus more susceptible to desiccation. *Zophosis amabilis* has a less permeable cuticle than that of *O. plana* and the lowest rate of water loss per unit surface area of the Namib Desert beetles investigated [26]. Although it has a weak response to fog events this species is not known to be a fog-harvester [40]. *Metriopus depressus* has not been observed to drink fog droplets [40] although is very active after rain and fog events [25]. This species exhibited a variation in the discontinuous gas exchange cycles where the mesothoracic spiracles exhibited a considerable shorter burst period and longer closed period when some of the CO_2_ was expelled through the elytral case. Further investigation is needed to determine whether the microtrichia fields could physically inhibit water leaving the subelytral cavity when the elytral case is partially opened. These beetles are known to retreat into crevasses in the rocks [25] and often with several individuals in a small space (JH Henschel, personal communication). By forming these aggregations these beetles potentially reduce their body water loss as was found in aggregations of *Parastizopus armaticeps*, a Kalahari Desert beetle [46]. Thus these beetles reduce water loss using a behavioural strategy in addition to physiological strategies.

## 5. Conclusions

All three ultraxerophilous tenebrionid species are active during the day on the sand surface away from vegetation where they are exposed to high temperatures and low humidity. The differences in their respiratory physiology can be linked to their ecology and behaviour. For the two small beetles inhabiting the gravel plain, conservation of water is important as neither species have been reported to drink fog although *M. depressus* activity increased after fog events and the *Z. amabilis* population shows a weak response to fog [47]. Both species reduced their respiratory water loss by using the mesothoracic spiracles as the main or sole site for gas exchange with a sealed subelytral cavity and increased the convection based flutter period. Interestingly *Z. amabilis* is a truly arid adapted species despite its small size as this beetle was present during the years without rainfall events when the populations of other species dropped [30]. *Metriopus depressus* also aggregates in rock crevasses as a possible behavioural means to reduce water loss. *Onymacris plana*, most abundant beetle species on the dune slipface, is a fog harvester and has several physiological mechanisms to cope with desiccation. This beetle is thus less water stressed than the other two species and uses all its spiracles for gas exchange.

## Figures and Tables

**Table 1:** Rate of CO_2_ emission in three Namib Desert tenebrionid beetles, including the separation of emission from pair of mesothoracic spiracles and elytral case. (mean ± SD).

Species	*Onymacris Plana*		*Metriopus Depressus*	*Zophosis Amabilis*
	Male	Female		
N	9	6	7	8
Mass (g)	0.813 ± 0.19	0.893 ± 0.39	0.214 ± 0.029	0.134 ± 0.04
Total rate of CO_2_ emission				
(µL h^−1^)	88.41 ± 17.13	97.38 ± 47.72	31.42 ± 16.23	22.26 ± 3.97
(µL h^−1^g^−1^) ^a^	118.72 ± 45.3	110.72 ± 37.74	146.95 ± 82.53	183.56 ± 61.54
Rate of CO_2_ emission (µL h^−1^)				
Mesothoracic spiracles	10.01 ± 11.04	28.62 ± 22.22	17.8 ± 13.62	18.51 ± 4.19
Elytral case	78.4 ± 24.76	68.76 ± 47.14	15.88 ± 7.88	4.98 ± 4.21
% total CO_2_ emitted through	13.28 ± 15.9	33.4 ± 28.8	57.4 ± 29.9	84.4 ± 18.4
mesothoracic spiracles				
DGC ^b^ frequency (cycles per hour)	8.94 ± 3.31	8.79 ± 4.35	9.62 ± 3.95	15.93 ± 5.97

^a^ Mass specific metabolic rate. ^b^ Discontinuous gas exchange cycles.

**Table 2:** Characteristics of the discontinuous gas exchange from the mesothoracic spiracles and elytral case of three Namib Desert tenebrionid beetles (mean ± SD).

Species	*Onymacris Plana*		*Metriopus Depressus*	*Zophosis Amabilis*
	Male	Female		
Flutter period:				
V_CO2_ (µL g^−1^) Mesothoracic spiracles	0.202 ± 0.19	0.842 ± 0.69	2.265 ± 1.791	2.429 ± 1.35
V_CO2_ (µL g^−1^) Elytral case	1.681 ± 0.81	1.599 ± 1.0	3.901 ± 1.441	0.406 ± 0.268
Ratio of Volume CO_2_				
mesothoracic spiracles: elytral case	0.19 ± 0.23	1.9 ± 3.1	0.52 ± 0.52	9.9 ± 9.7
Duration (s)	211.9 ± 123	274.3 ± 166	439.8 ± 93	142.8 ± 69
% Flutter period of DGC ^a^	42.1%	43.1%	68.3%	53.5%
Burst period:				
V_CO2_ (µL g^−1^) Mesothoracic spiracles	1.35 ± 1.65	3.95 ± 3.89	7.364 ± 4.01	7.995 ± 2.52
V_CO2_ (µL g^−1^) Elytral case	10.019 ± 3.42	7.143 ± 4.73	6.592 ± 3.23	1.721 ± 1.53
Ratio of volume CO_2_				
mesothoracic spiracles: elytral case	0.24 ± 0.38	1.91 ± 3.32	2.0 ± 1.8	18.4 ± 22.9
Duration (s)	138.9 ± 59	202.6 ± 192	129.1 ± 20	71.1 ± 19
% Burst period of DGC ^a^	31.3%	31.4%	30%	29.8%

^a^ Discontinuous gas exchange cycles.

## Data Availability

Original data is available from the author on request.
